# How the pandemic affected the frequency, type and intensity of migraines in students

**DOI:** 10.51866/lte.741

**Published:** 2024-11-11

**Authors:** Sinda Zarrouk, Josef Finsterer

**Affiliations:** 1 MD, PhD, Neurology Dpt., Neurology & Neurophysiology Center, Vienna, Austria. Email: fifigs1@yahoo.de; 2 PhD, University of Tunis El Manar and Genomics Platform, Pasteur Institute of Tunis, Tunisia.

**Keywords:** Headache, Pandemics, SARS-CoV-2, Questionnaire, Migraine disorders

**Dear editor,**

We read with interest the article by Selvakumar et al. on the prevalence of migraine in students.^1^ Their cross-sectional study was conducted from December 2021 to April 2022 using an online questionnaire designed by the investigating authors. The prevalence of migraine in the cohort studied was 35.9%. The most common triggers of migraine were stress and a lack of sleep, and the most commonly used coping strategy was lifestyle changes. None of the triggers, coping strategies and clinical features of migraine were correlated with COVID-19. The study is excellent, but some points should be discussed.

The first point relates to the use of an online questionnaire. Online questionnaires have several disadvantages. First, it cannot be guaranteed that the respondent is the person answering the questions. Second, it is not easy to check whether the answers given are correct and true. Third, additional questions that might arise in a conversation might not come up and would not be asked. Fourth, it cannot be ensured that the respondent is cognitively capable of understanding the questions and answering them appropriately or physically capable of operating the electronic device.

The second point is that it is unclear whether all included patients had their onset of migraine before the pandemic or whether patients who developed migraine during the pandemic were also included. This is important, as there are several reports of patients developing migraine in temporal relation to SARS-CoV-2 infection.^2^ In addition, migraine following SARS-CoV-2 infection may manifest differently from migraine due to other causes.

The third point is that the difference between the ‘severity’ and ‘intensity’ of migraine as mentioned in Table 2 is unclear. The authors need to clarify how they differentiated between these two characteristics and how the respondents were expected to distinguish between them. The patients may have answered ‘yes’ or ‘no’ to both questions because they could not differentiate between the two characteristics.

The fourth point is that the study did not report how many patients were vaccinated and how many were not. This is important because SARS-CoV-2 vaccination can trigger new-onset migraine^3^ or worsen the intensity and frequency of existing migraine.^4^

The fifth point is that no control group of patients with migraine from outside the pandemic period was included and evaluated using the same questionnaire. Therefore, it is not possible to assess whether the specific situations of the pandemic increased, decreased or left unchanged the frequency, type and intensity of migraine.

The sixth point is that the data were not analysed according to migraine type. It is essential to know how many of the included patients experienced migraine with or without aura, chronic migraine, vestibular migraine, abdominal migraine, hemiplegic migraine, menstrual migraine or migraine with brainstem aura and whether these different types occurred with a different frequency compared to the non-COVID-19 period.

The seventh point is that the study did not report or analyse how many of the included patients had contracted SARS-CoV-2 infection and to what extent the infection influenced the characteristics of their migraine.

In summary, this interesting study has limitations that contextualise its results and their interpretations. Addressing these limitations could strengthen the conclusions and corroborate the message of the study. The intensity, type and frequency of migraine during the pandemic should be assessed through on-site investigations, with the inclusion of an appropriate control group to obtain reliable results.
